# Taxonomic and Functional Compositions Impacted by the Quality of Metatranscriptomic Assemblies

**DOI:** 10.3389/fmicb.2018.01235

**Published:** 2018-06-20

**Authors:** Maggie C. Y. Lau, Rachel L. Harris, Youmi Oh, Min Joo Yi, Aida Behmard, Tullis C. Onstott

**Affiliations:** ^1^Department of Geosciences, Princeton University, Princeton, NJ, United States; ^2^Program in Atmospheric and Oceanic Sciences, Princeton University, Princeton, NJ, United States; ^3^Department of Ecology and Evolutionary Biology, Princeton University, Princeton, NJ, United States; ^4^Department of Astrophysical Sciences, Princeton University, Princeton, NJ, United States

**Keywords:** RNA-Sequencing, *de novo* metatranscriptomics, taxonomic composition, metabolic functions, metaproteomics

## Abstract

Metatranscriptomics has recently been applied to investigate the active biogeochemical processes and elemental cycles, and *in situ* responses of microbiomes to environmental stimuli and stress factors. *De novo* assembly of RNA-Sequencing (RNA-Seq) data can reveal a more detailed description of the metabolic interactions amongst the active microbial communities. However, the quality of the assemblies and the depiction of the metabolic network provided by various *de novo* assemblers have not yet been thoroughly assessed. In this study, we compared 15 *de novo* metatranscriptomic assemblies for a fracture fluid sample collected from a borehole located at 1.34 km below land surface in a South African gold mine. These assemblies were constructed from total, non-coding, and coding reads using five *de novo* transcriptomic assemblers (Trans-ABySS, Trinity, Oases, IDBA-tran, and Rockhopper). They were evaluated based on the number of transcripts, transcript length, range of transcript coverage, continuity, percentage of transcripts with confident annotation assignments, as well as taxonomic and functional diversity patterns. The results showed that these parameters varied considerably among the assemblies, with Trans-ABySS and Trinity generating the best assemblies for non-coding and coding RNA reads, respectively, because the high number of transcripts assembled covered a wide expression range, and captured extensively the taxonomic and metabolic gene diversity, respectively. We concluded that the choice of *de novo* transcriptomic assemblers impacts substantially the taxonomic and functional compositions. Care should be taken to obtain high-quality assemblies for informing the *in situ* metabolic landscape.

## Introduction

Metatranscriptomics has been applied to investigate environmental microbiomes in recent years (e.g., Stewart et al., 2011; Jiang et al., 2012; Tveit et al., 2013; Baker et al., 2013; Hultman et al., 2015; Lau et al., 2015, 2016; Fortunato and Huber, 2016). Compared to analyzing DNA sequences from a mixed-species community (metagenomics), the analysis of total cellular RNA sequences can distinguish active members from inactive microbial members, and can identify functional traits that the active members likely exhibited under *in situ* conditions. However, it remains challenging to fully exploit the massive information stored in the short RNA sequences generated by next-generation sequencing platforms (Schliesky et al., 2012). If we could extract more information from metatranscriptomic data, it will greatly enrich our knowledge in geobiology and microbial ecology by adding insights into, for instance, the active biogeochemical processes and elemental cycles, as well as microbial responses to environmental stimulants and stress factors.

RNA-Sequencing (RNA-Seq) has proven to be a powerful tool in cataloging transcriptomes in samples due to its high throughput at relatively low cost and low background noise compared to Sanger sequencing and hybridization-based technologies (reviewed by Wang et al., 2009). *De novo* transcriptomic assemblies for isolated single species have gained much attention, as shown by the rapid development and usage of many open-source assembly programs in the past 5 years equipped to handle high-volume RNA-Seq data (e.g., Robertson et al., 2010; Grabherr et al., 2011; Bankevich et al., 2012; Schulz et al., 2012; Peng et al., 2013; Tjaden, 2015) that span a dynamic range of 10^2^ to 10^5^ copies per transcript. However, this approach has not yet been widely applied to metatranscriptomics in environmental microbiology studies; and when applied, the quality of these assemblies has not been thoroughly assessed.

We define the metabolic landscape of an environment as a representation of the metabolic network utilized by participating active taxonomically and functionally diverse microorganisms over space and time. We also define the quality of a *de novo* metatranscriptomic assembly by how well it renders taxonomic and functional compositions of an active community, and therefore the comprehensiveness of the resultant metabolic landscape of the studied environment. This evaluation relies on the number of transcripts, transcript length, range of transcript coverage, continuity, and percentage of transcripts with confident annotation assignments, taxonomic and functional diversity patterns, and functional inference validated by metaproteomic data. This study aimed to compare the quality of *de novo* assemblies generated by five *de novo* metatranscriptomic assemblers, namely Trans-ABySS, Trinity, Oases, IDBA-tran, and Rockhopper. The best-performing assembler for coding and non-coding reads was identified based on the criteria outlined above.

## Materials and methods

### RNA-sequencing and proteomic datasets

Fracture water was collected in 2012 from a borehole in the Beatrix gold mine (shaft No. 3 Level 26) located SW of South Africa's Witwatersrand basin. A detailed description of the study site, sample collection and sample extraction protocol of this sample, BE326FW270712 Bh2 (BE2012), has been published in Lau et al. (2014), Magnabosco et al. (2014), and Lau et al. (2016). In brief, the valve that sealed the borehole from the ambient tunnel conditions was opened to allow the fracture water to flow through a sterile stainless-steel manifold for several minutes. The water flowed at > 50 L per min, flushing out fracture water near the borehole opening that might have been contaminated by tunnel air, and preventing oxygen from getting into the borehole. A massive filter contained in a stainless-stain housing was left onsite for 15 days to accumulate sufficient biomass. Upon collection, the filter was immediately preserved in RNA-preservation solution (Lau et al., 2016), and then stored frozen at −80°C until extraction. The topic of potential contamination is discussed in the Supplementary Text.

RNA and proteome samples were extracted in parallel with DNA from the filter sample using phenol/chloroform. Total RNA was purified by ethanol precipitation and subsequently treated with DNase I (Sigma-Aldrich). PCR amplification of the treated RNA yielded no PCR products after 35 cycles, indicating the sample was DNA-free. Methodology of directional RNA-Sequencing and quality-filtering has been described in Lau et al. (2016). Briefly, Illumina 2500 HiSeq platform generated 29,980,240 single-end reads of 141-nt in length. Reads with 90% of the bases with Q score ≥30, without adaptor sequences, and without ambiguous bases were kept for analysis.

As described in Lau et al. (2016), total protein was purified by methanol/acetone purification, fractionated, trypsin-digested, and subsequently analyzed using ultrahigh-performance liquid chromatography-tandem mass spectrometry (UPLC-MS/MS). The 10 fractions of tandem MS/MS data were aggregated and analyzed using the SEQUEST HT search engine in ProteomeDiscoverer v1.4 (Thermo Fisher Scientific). All predicted protein-encoding genes (PEGs) in the five coding RNA (cRNA) assemblies were compiled into a single protein database. This protein database differs from that used by Lau et al. (2016), which was constructed from Trinity cRNA assembly alone. [How coding and non-coding RNA (ncRNA) were separated is explained in the next section]. The search criteria described in Lau et al. (2016) were adopted to identify PEGs. Expression of a PEG in the metaproteomic data was considered valid (Lau et al., 2016): (i) if the PEG was identified by matches to at least two unique peptides in a single or replicated UPLC-MS/MS runs; or (ii) if it was identified only in one run and by a single unique peptide, but at least five peptide spectral matches (PSMs) were assigned to the PEG.

The raw RNA-Seq data have been made available at the National Center for Biotechnology Information (BioProject ID: PRJNA308990; BioSample ID: SAMN04419122; Lau et al., 2016). The raw mass spectrometry proteomics data have been deposited at the ProteomeXchange Consortium via the PRIDE partner repository (dataset identifier PXD004634; Lau et al., 2016).

### *De Novo* metatranscriptome assembly

**Figure 1:**
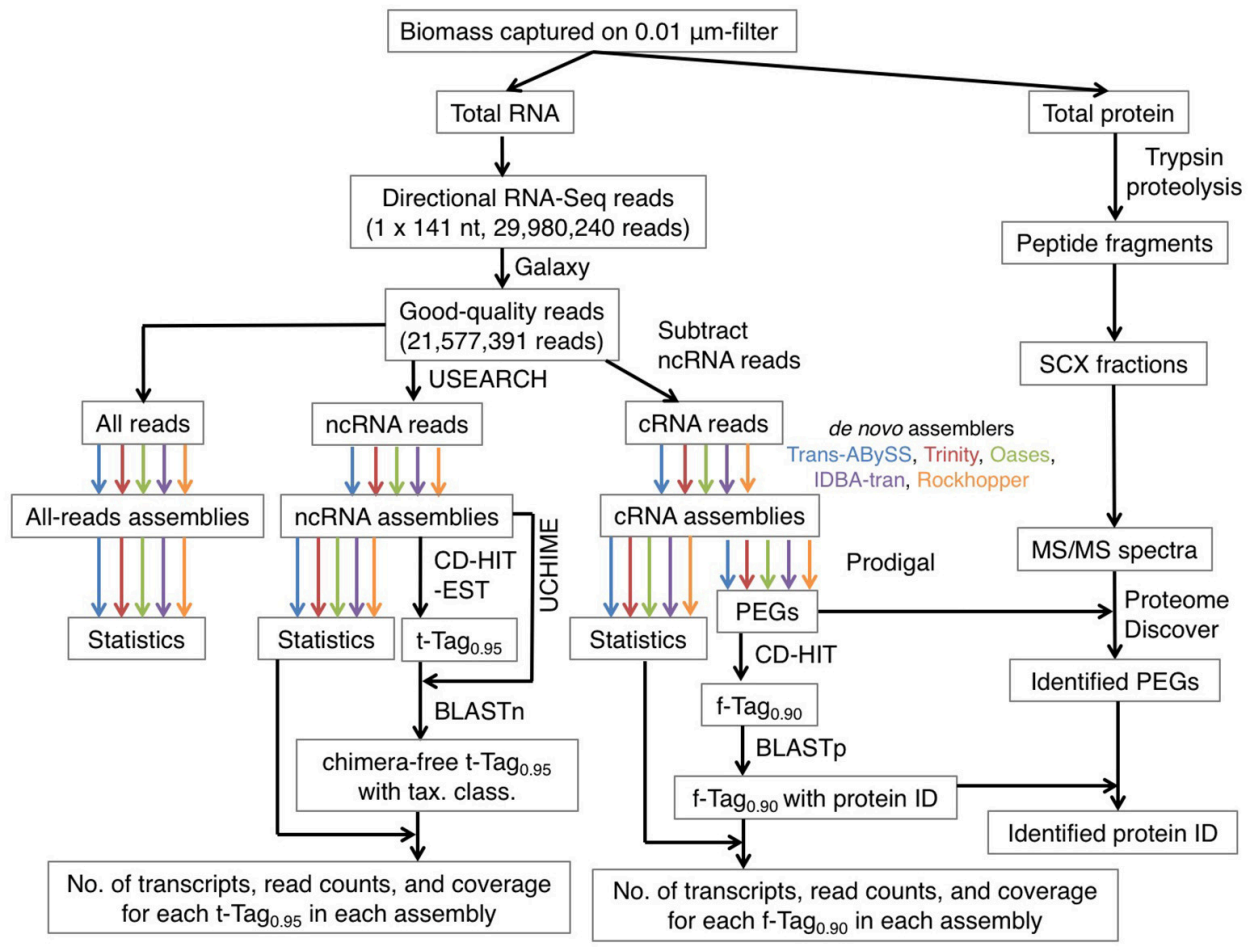
An overview of the bioinformatics workflow. The color arrows indicate the generation of 15 assemblies using five *de novo* transcriptomic assemblers, namely Trans-ABySS (blue), Trinity (red), Oases (olive), IDBA-tran (purple), and Rockhopper (orange). Abbreviations: ncRNA, non-coding RNA; cRNA, coding RNA; tax. class., taxonomic classification; PEG, protein-encoding gene; protein ID, protein identity; SCX, strong cation exchange; MS/MS, tandem mass spectrometry.

To test whether dividing the RNA reads into protein-coding and non-coding sequence pools would enhance assembly quality, the quality-filtered (hereafter, good-quality) RNA-Seq reads were organized into two subsets, coding RNA (cRNA) sequences and non-coding RNA (ncRNA) sequences (Figure 1). All good-quality RNA reads were searched against four databases using USEARCH (Edgar, 2010) for its shorter run-time comparing to BLAST, as described in Lau et al. (2016). The four databases (DB) were transfer RNA DB (Abe et al., 2011, 2014; 872,667 sequences), 5S ribosomal RNA (rRNA) DB (Szymanski et al., 2002; last updated in Sept, 2005; 1,379 sequences), small and large rRNA DBs (Quast et al., 2013; version 119.1; 4,346,329 sequences in SSUParc_tax_silva.fasta.gz and 446,998 sequences in LSUParc_tax_silva.fasta.gz). RNA reads were assigned into the ncRNA subset when they shared at least 80% of identity in global alignment with any of references in the four DBs, regardless of the orientation. The options used were “-usearch_global,” “-uid 0.8,” “-strand both,” and “-threads” for multithreading. The remaining RNA reads were assigned into the cRNA subset. A python script was used to parse the total RNA reads into cRNA and ncRNA subsets, which contain 1,498,563 (7%) and 20,078,828 (93%) reads respectively.

*De novo* assembly was then performed on all RNA reads, cRNA and ncRNA subsets separately (Figure 1). Five transcriptomic assemblers were tested, namely Trans-ABySS (Robertson et al., 2010), Trinity (Grabherr et al., 2011), Oases (Schulz et al., 2012), IDBA-tran (Peng et al., 2013), and Rockhopper (Tjaden, 2015). These license-free assemblers use substrings of reads (k-mers) and *de Bruijn* graphs to construct contigs. However, each assembler is designed to address different problems that transcriptomic data pose to assembly, as reviewed by Martin and Wang (2011). Thus, each has adopted different sorting, clustering, pruning, merging, and information-storing strategies to construct and examine possible paths before outputting the final set of contigs. We recommend referring to the original articles for the details of each algorithm. Presented here is how each assembler was applied to our RNA-Seq data (Table 1).

**Table 1:** Summary of strategies used for generating metatranscriptomic assemblies.

	**Trans-ABySS**	**Trinity**	**Oases**	**IDBA-tran**	**Rockhopper**
Version	1.5.2	r20131110	0.2.0	1.1.0	2.0
Assembly method	*De novo assembly* using *de Bruijn graph*				
k-mer size (min, max, step)	61, 101, 4	25	61, 101, 4	61, 101, 4	25
Minimum contig size	200 bp	200 bp	200 bp	300 bp	200 bp
Option “strand-specific” specified?	Yes	Yes	Yes	Not available	Yes
No. of intermediate assemblies generated	11	1	11	11	1
Merging of assemblies	Pairs of assemblies with adjacent k values were reciprocally compared to find representative contigs. Representative contigs were the longest parent contig to which the shorter contigs had an exact match. The merging step was reiterated.	No applicable	Contigs in the 11 assemblies were used as input for assembly in Velvet. The k-mer (or K_MERGE_) size of 27, options “-long,” “-conserveLong,” and “-read_trkg” were used. Identical graphs across the 11 assemblies were removed.	Assemblies were generated reiteratively with the contigs generated in the previous assembly being used to generate k-mers for the next assembly.	No applicable

All input files used were in fasta format. For all assemblers, the first step is to create a k-mer hash table. The strand-specific option, if available, was indicated in the execution command. Contigs in the final assemblies have a minimum length of 200 and 300 nt for IDBA-tran.

- Trans-ABySS (version 1.5.2; Robertson et al., 2010): Contigs were constructed from k-mer size = 61–101, with 4-mer step increments, as separate assemblies using ABySS (version 1.5.2) (Simpson et al., 2009). For each assembly, overlapping k-mers were used to build *de Bruijn* graphs. Erroneous parts were removed to simplify the paths and create contigs, which were linked as guided by the original, good-quality RNA-Seq reads. Pairs of assemblies with adjacent k values were reciprocally compared to find representative contigs. Representative contigs were the longest parent contig to which the shorter contigs had an exact match. The merging step was reiterated to obtain a single, non-redundant set of contigs.
- Trinity (version r20131110) (Grabherr et al., 2011): The default (and non-adjustable) k-mer size was 25. Contigs were extended from both ends of the “seed” sequence by a greedy approach of (k-1) overlap, and the longest contig sequence generated from each “seed” was kept. Contigs that shared common region(s) were clustered. *De Bruijn* graphs were then drawn for each cluster. The original information present in good-quality RNA-Seq reads was used to simplify and verify the paths in order to decompose the *de Bruijn* graphs into the final set of contigs.
- Oases (version 0.2.0) (Schulz et al., 2012): Contigs were constructed from k-mer size = 61–101, with 4-mer step increments, yielding 11 assemblies using Velvet version 1.2.10 (Zerbino and Birney, 2008). For each assembly, overlapping k-mers were used to build *de Bruijn* graphs (Velveth and Velvetg in Velvet). Oases reiteratively visited the parallel paths for contig correction. After correction, contigs were connected by referencing the good-quality RNA-Seq reads and subsequently organized them into clusters representing different loci. Redundant connections among contigs within each locus cluster were removed before the graphs were decomposed into contigs. Contigs in the 11 assemblies were used as input for assembly in Velvet. The k-mer (or K_MERGE_) size of 27, as recommended by the authors, and option “-long” were used to build the hash table for each assembly. Options “-conserveLong” and “-read_trkg” were then used at the graph-traversing step. Identical graphs across the 11 assemblies were removed during the merging step to reduce the redundancy in the final set of contigs.
- IDBA-tran (version 1.1.0) (Peng et al., 2013): Contigs were first constructed from k-mer size = 61. Paths were pruned based on a probability model built from the input dataset, and verified by referencing to the good-quality RNA-Seq reads. The contigs generated were broken into k-mer size = 71, and the k-mers were then used as input for assembly. The pruning and validation processes were repeated for larger k-mer sizes with the maximum k-mer size = 101, at an increment of 10.
- Rockhopper (version 2.0) (Tjaden, 2015): The default k-mer size was 25. K-mers were used to build a *de Bruijn* graph, which was turned into candidate contigs stored in a Burrows Wheeler Index (BWI). If the k-mer already existed in the contigs, it was ignored; otherwise, the *de Bruijn* graph and BWI were updated. Good-quality RNA-Seq reads were mapped against the candidate contigs to finalize the assembly.

From this point forward, contigs are referred to as transcripts. These transcripts were partial or near-complete in length. In total, 15 assemblies of transcripts were generated from the three datasets (all good-quality RNA-Seq reads, ncRNA reads, and cRNA reads) by the five *de novo* assemblers. Mean base coverage for each transcript was calculated by first multiplying the number of quality-filtered reads mapped to the transcript by the average read length, and then this value was divided by the transcript length, as described in Lau et al. (2016).

### Quality assessment of the assemblies

Basic statistics were computed using a python script for each assembly. They included the number of transcripts ≥200 nt, total assembled length, maximum transcript length, minimum transcript length, average transcript length, the N50 and N90 lengths and the number of transcripts contributed to these two indices.

To evaluate a transcriptomic assembly originating from an isolate for which well-annotated complete genome sequences exist, the genome sequences serve as a benchmark. For samples that have incomplete genomic record such as environmental samples containing uncultivated species, there is no reliable benchmark dataset available to assess the quality of metatranscriptomic assemblies. Hence, we evaluated the representativeness of assembled transcripts by mapping the read datasets back to the corresponding assemblies generated from the read datasets. The mapping rate was estimated using Bowtie2 version 2.2.5 (Langmead and Salzberg, 2012), options “–end-to-end” for global alignment, “–norc” for known strand-specificity and “–very-sensitive” mode for higher accuracy.

Assemblies generated from each of the three datasets were also evaluated using RSEM-EVAL (RNA-Seq by Expectation Maximization-EVALuation) in the DETONATE (*DE novo* TranscriptOme rNa-seq Assembly with or without the Truth Evaluation) package (Li et al., 2014). RSEM-EVAL constructs a customized probabilistic model for an assembly based on the number of reads, the number of contigs and read coverage. The scores provide a quantitative basis to select the best assembly for RNA-Seq data, for which an ideal reference does not exit (Li et al., 2014). The RSEM-EVAL score imposes a parsimony preference in that a greater percentage of RNA-Seq reads is represented by fewer number of contigs. The lengths of coding genes in 30 published genomes, representing 30 genera that have been detected in the crustal fluids in South Africa (Lau et al., 2014), was used to calculate the expected transcript length distribution. The average length and standard deviation for all genes, protein-coding genes alone, and ribosomal RNAs alone were determined to be 985.29 ± 709.53, 944.2 ± 709.70, and 2,216.95 ± 703.60 nt, respectively. These values were used in the calculation of RSEM-EVAL scores for the 15 assemblies. Alignment was performed using the “very_sensitive” mode of Bowtie2.

Additionally, assemblies were compared to determine the differences in the taxonomic and functional compositions of the mixed-species community. Transcripts of ncRNA, primarily rRNA genes, generated from different assemblers were clustered using CD-HIT-EST in the CD-HIT package version 4.6.4 (Fu et al., 2012), at an identity cutoff level of 95% (word size “-n” = 10). The representative sequences were searched against the rRNA database that was comprised of SILVA small and large rRNA gene sequences, or SSU and LSU, respectively (version 119.1). Taxonomic assignment was based on the taxonomic classification of their BLASTn results. Ten best hits were collected, and those that aligned ≥50% of the query sequence, had an e-value of <1e-5 and a bitscore of ≥50 were considered, or else, discarded. The query sequence was assigned with the consensus rRNA identity (SSU or LSU) by majority rule, and taxonomic rankings by the lowest common ancestor (LCA) principle (threshold = 80%). The same rRNA database was used to identify chimeric rRNA gene sequences using UCHIME (Edgar et al., 2011). Transcripts were clustered into operational taxonomic units at 95% identity (t-Tag_0.95_), and being conservative, any ncRNA transcripts in clusters represented by chimeras were omitted from further analysis.

PEGs were predicted from transcripts generated from the cRNA dataset. Prodigal version 2.6.1 (Hyatt et al., 2010) was used to identify open reading frames using the option “-p” for metagenome, and the translation table 11. As the orientation of the RNA-Seq reads was known and all the transcripts ran in the sense direction, any predictions made for the anti-sense orientation were discarded. A python script was used to extract the amino acid sequences translated from the sense strands. The similarity of the peptides generated from different assemblers was compared using CD-HIT. Peptides were clustered at 90% of identity (word size “-n” = 5) for local alignment (“-G” = 0), using a slow mode (“-g” = 1) such that the query sequence would be clustered into the most similar cluster that met a threshold defined by 80% alignment of shorter sequences to the respective representative sequences (“-aS” = 0.8). The longest sequence in each cluster (f-Tag_0.90_) was searched against the NCBI non-redundant protein (nr) database using BLASTp. Ten best hits were collected, and those that aligned ≥50% of the query sequence, had an *e*-value of ≤1e-5 and a bitscore of ≥50 were considered, or else discarded. Finally, the query sequence was assigned by majority rule with the consensus protein identity based on the description of entries in the NCBI nr database.

### Availability of scripts and assembled data

The workflow has been segmented into sections and wrapped into shell scripts to make it more user friendly. Details of how to implement and customize the workflow are available in Supplementary Materials File 1, and on GitHub (https://github.com/maglau/De-novo-metatranscriptomic-analysis-workflow). The representative sequences of transcripts generated from cRNA (Supplementary Materials File 2) and ncRNA (Supplementary Materials File 3) reads are also included in Supplementary Materials. Transcripts of individual 15 assemblies will be made available upon request.

## Results

### Number of transcripts and their expression levels

The total number of transcripts assembled varied by three orders-of-magnitude, ranging from hundreds to tens of thousands of transcripts per assembly (Table S1). Trans-ABySS assembled the most number of transcripts for ncRNA and total RNA datasets, i.e., 12,783 ncRNA transcripts and 23,180 transcripts, respectively. However, the total length of these two assemblies (4.9 Mbp in the ncRNA assembly, and 8.4 Mbp in the total RNA assembly) was shorter than that of the Oases ncRNA (6.3 Mbp) and total RNA (8.5 Mbp) assemblies. Trinity generated the greatest number of transcripts (14,678) and the greatest total assembled length (5.1 Mbp) for the cRNA dataset. Whereas Rockhopper generated the least number of transcripts and the least total assembled length for all three datasets.

**Figure 2:**
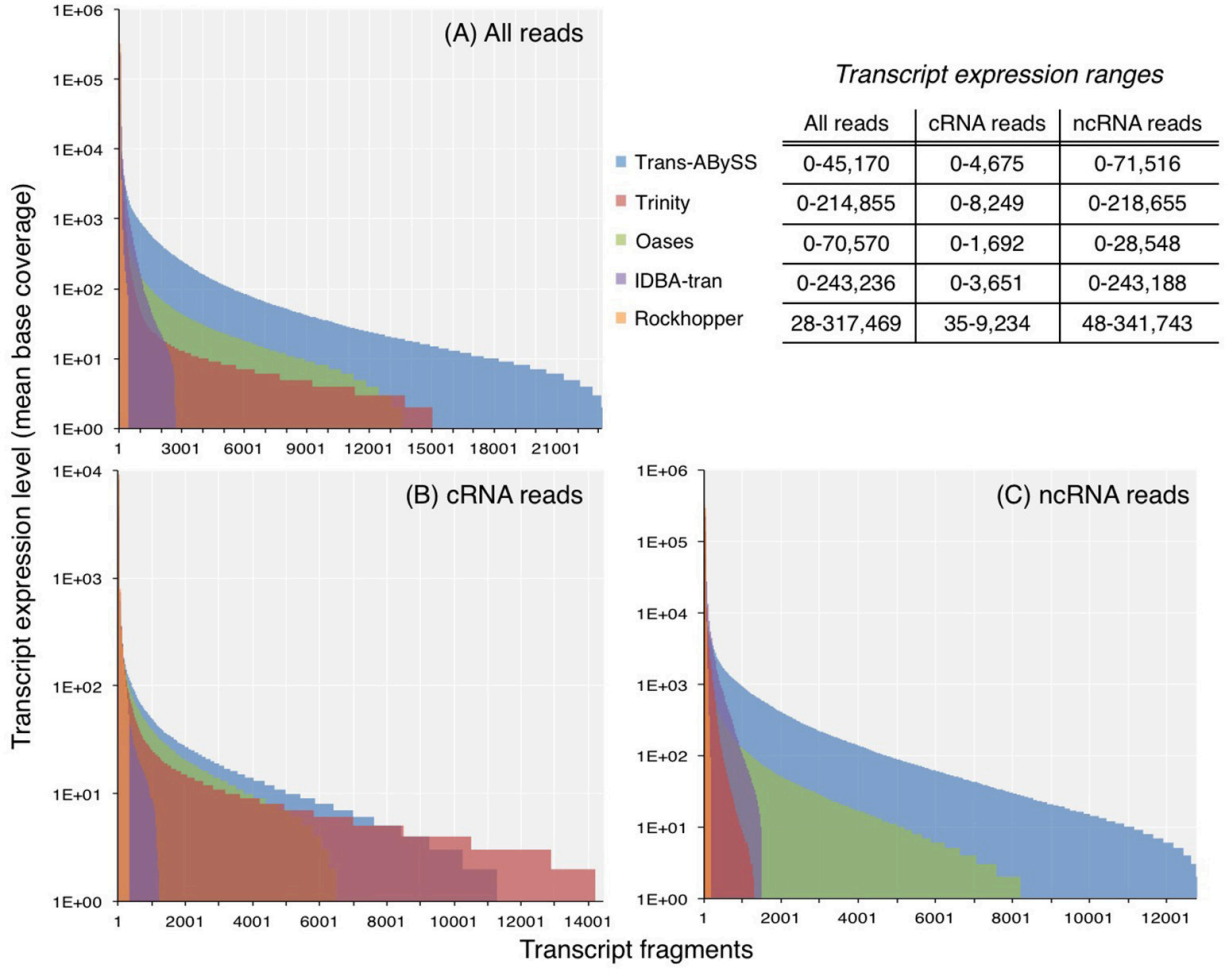
Expression levels of transcripts in assemblies generated from **(A)** all reads, **(B)** coding RNA reads, and **(C)** non-coding RNA reads. Coverage was computed by normalizing read counts, that mapped to the transcript, to its length. For transcripts with zero coverage, log(coverage+1) was plotted.

The ncRNA and total RNA assemblies showed a greater expression range than the cRNA assemblies (Figure 2). Transcripts in ncRNA and total RNA assemblies showed an expression level up to 10^5^-10^6^, whereas the maximum expression level estimated for cRNA transcripts were at least an order of magnitude lower. Although the expression ranges in all the assemblies spanned across few orders-of-magnitude, the majority of transcripts had a mean base coverage of 200 times or less (Figures 2A–C).

### Transcript length and continuity

**Figure 3:**
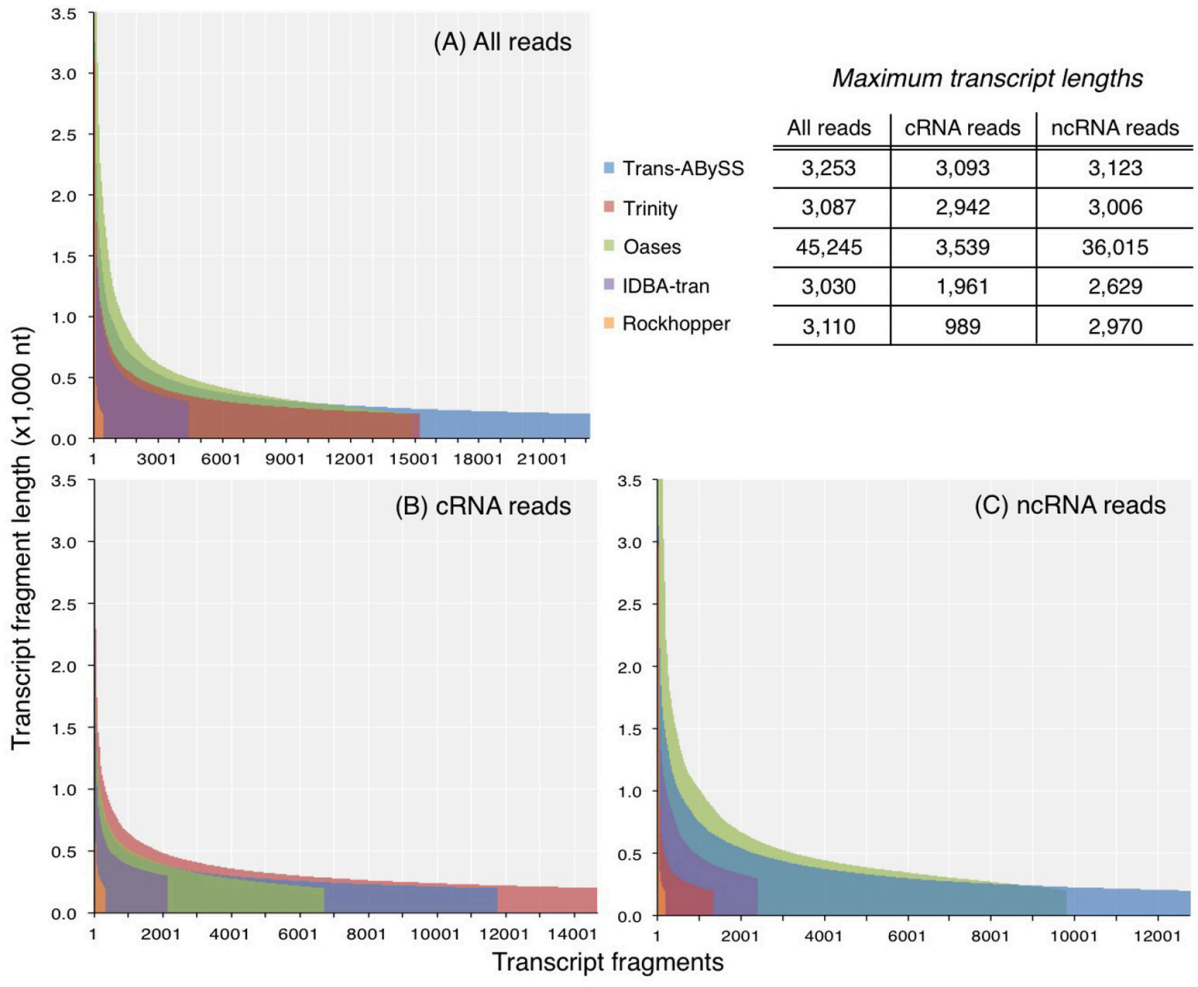
Length distribution of transcripts in assemblies generated from **(A)** all reads, **(B)** coding RNA reads, and **(C)** non-coding RNA reads. Y-axis was truncated in **(A)** and **(C)** to visualize the shorter transcripts, which dominated the assemblies.

The N50 values of ncRNA, cRNA, and total RNA assemblies were 297–746, 296–417, and 309–645 nt. N90 values exhibited a narrower range, from 214 to 334 nt (Table S1). For each of the three datasets, Oases created the longest transcripts of all assessed assemblers (Table S1; Figure 3). Published sequences of large subunit of rRNA (LSU) genes are on an average about 3,000 bp long; however, about 1% of the Oases ncRNA transcripts, comprised of small subunit of rRNA (SSU), were 10 times longer than 3,000 bp. A BLASTn search of the five longest Oases ncRNA transcripts (33,602–36,015 nt) revealed that the top hits were prokaryotic LSU genes, yet there were multiple alignment regions along the Oases ncRNA transcripts, meaning the exceptionally long Oases ncRNA transcripts contained fragmented LSU signatures. Similarly, from the total RNA dataset, which was dominated by ncRNA reads (93% of all good-quality RNA-Seq reads), Oases also assembled extraordinarily long transcripts (Table S1, Figure 3). These long transcripts also contained fragmented LSU signatures. The presence of these suspicious transcripts explained the very low mapping rates when compared to other total RNA and ncRNA assemblies (Table S1). These results clearly indicated that Oases has problem assembling ncRNA genes in metatranscriptomic data. Though Rockhopper generated the smallest assemblies, the range of transcript length (Figure 3) and the percentage of mapped reads were comparable to the larger assemblies (Table S1). Considering the speedy runtime (less than 5 min per run) of Rockhopper, these results showed that the simple, straightforward assembly algorithm is in fact quite effective and efficient. Its competence would increase if the *de Bruijn* graph network and information storage scheme could be modified to resolve more transcripts.

**Figure 4:**
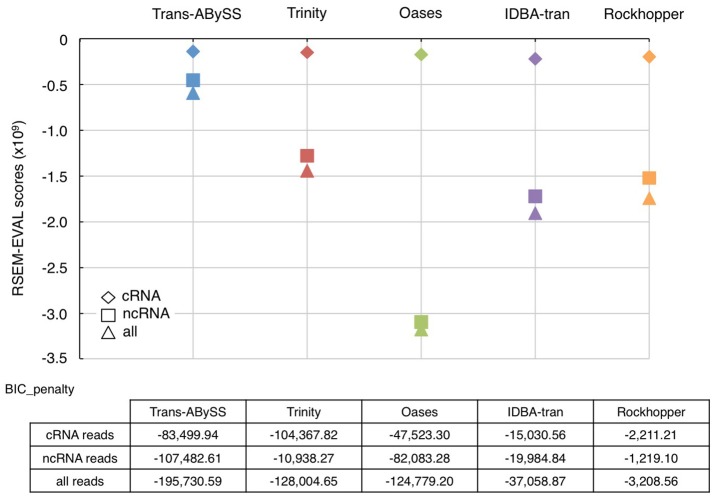
RSEM-EVAL scores and BIC penalties of metatranscriptomic assemblies. The RSEM-EVAL scores computed for the assemblies generated from cRNA (diamond), ncRNA (square), and all RNA (triangle) reads by the five novo transcriptomic assemblers were indicated using the same color scheme as in Figure 1. The closer is the RSEM-EVAL score to zero, the better the assembly. The complexity of these assemblies was reflected by the Bayesian information criterion (BIC) penalty score. An assembly's complexity is assumed to be inversely proportional to its size.

As an ideal reference does not exist for environmental samples, RSEM-EVAL was used to evaluate continuity (Li et al., 2014). The RSEM-EVAL scores are negative; the closer it is to zero, the better the assembly. All assemblies showed an RSEM-EVAL score within the same order-of-magnitude as the values reported for simulated and empirical datasets (−2 × 10^9^) (Li et al., 2014). The RSEM-EVAL scores for the cRNA assembly, the ncRNA assembly, and the assembly generated using all RNA-Seq reads were increasingly negative for all assemblers (Figure 4). This trend followed the dataset size because the scoring model has a Bayesian information criterion (BIC) term that penalizes proportionally with increasing size of dataset (Li et al., 2014). The RSEM-EVAL scores for the five cRNA assemblies were very similar, with those assembled by Trans-ABySS and Trinity being higher (i.e., less negative). However, the RSEM-EVAL scores of ncRNA assemblies were drastically different among assemblers, and were very similar to the RSEM-EVAL scores of the corresponding assemblies generated from all RNA-Seq reads. This is likely because the total RNA-Seq reads were comprised of ~93% of ncRNA reads. Trans-ABySS and Trinity generated ncRNA assemblies with greater continuity than the remaining three assemblers. Oases ncRNA and total RNA assemblies yielded the most negative RSEM-EVAL scores, implying high discontinuity. This is in agreement with the observation of segmented genes in these transcripts, and the low number of mapped reads that were discussed earlier.

### Quality assessment based on homology search

By mapping all RNA-Seq reads to ncRNA and cRNA transcripts, some reads were found to align with both types of transcripts (data not shown). The complexity in the total RNA dataset might have generated error-prone transcripts that mistakenly joined ncRNA to cRNA reads (i.e., mis-assembly). In addition, although all assemblers generated more transcripts from the total RNA dataset than from the ncRNA or cRNA subsets (Table S1), the transcripts generated from the ncRNA and cRNA subsets together outnumber that from the total RNA, and have longer assembled length in total. As a result, ncRNA assemblies were analyzed for taxonomic composition, and cRNA assemblies were analyzed for functional composition.

A considerable number of ncRNA transcripts were identified as chimeras, ranging from 6% (Oases) to 27% (Trans-ABySS) (Table 2). Up to 23% of non-chimeric transcripts matched to none of the sequences in the SILVA rRNA database or had matches that did not meet the quality thresholds specified in the Methods (Table 2). These ncRNA transcripts were also discarded. It was noted that all long, dubious transcripts generated by Oases belonged to this low-quality bin.

**Table 2:** Summary of *de novo* metatranscriptomic assemblies of non-coding (nc) and coding (c) RNA-Seq reads based on homology search.

		**Trans-ABySS**	**Trinity**	**Oases**	**IDBA-tran**	**Rockhopper**
**ncRNA TRANSCRIPTS**						
1	Total number of transcripts	12,783	1,300	9,762	2,376	144
2	Number of chimeric sequences (% of row 1)	3,488 (27%)	217 (17%)	590 (6%)	358 (15%)	25 (17%)
3	Number of non-chimeric transcripts that has no or low confidence hit against SILVA rRNA database (% of row 1)	867 (6%)	185 (14%)	2,217 (23%)	15 (1%)	9 (6%)
4	Number of transcripts included for taxonomic composition analysis (% of row 1)	8,428 (66%)	898 (69%)	6,955 (71%)	2,003 (84%)	110 (76%)
5	Number of SSU transcripts ≥1,200 nt (% of row 4)	58 (0.6%)	0 (0%)	83 (0.9%)	32 (1.6%)	1 (0.8%)
6	Number of LSU transcripts ≥2,400 nt (% of row 4)	22 (0.2%)	3 (0.3%)	27 (0.3)	2 (0.1%)	2 (1.7%)
7	Number of t-Tag_0.95_ clusters (% of the grand total, i.e., 7,288)	4,506 (62%)	777 (11%)	2,949 (40%)	1,287 (18%)	90 (1%)
8	Number of consensus phyla (% of the grand total, i.e., 43)	39 (91%)	39 (91%)	39 (91%)	35 (81%)	17 (40%)
**cRNA TRANSCRIPTS**						
9	Total number of transcripts	11,743	14,678	6,683	2,113	310
10	Number of transcripts containing protein-encoding genes (PEGs) (% of row 9)	8,643 (74%)	13,509 (92%)	5,343 (80%)	1,829 (87%)	233 (75%)
11	Number of transcripts containing sense PEGs (% of row 9)	6,877 (59%)	12,319 (84%)	4,413 (66%)	964 (46%)	204 (66%)
12	Total number of PEGs	9,429	15,705	6,032	2,232	258
13	Number of PEGs in sense direction (% of row 12)	7,507 (80%)	14,197 (90%)	4,976 (82%)	1,130 (51%)	222 (86%)
14	Number of PEGs that has no or low confidence hit against NCBI NR database (% of row 12)	2,197 (23%)	2,802 (18%)	1,157 (19%)	246 (11%)	49 (19%)
15	Number of PEGs included for functional composition analysis (% of row 12)	5,310 (56%)	11,395 (73%)	3,819 (63%)	884 (40%)	173 (67%)
16	Number of PEGs within 95% of the length of their BLASTp best hits (% of row 15)	153 (3%)	461 (4%)	127 (3%)	38 (4%)	0 (0%)
17	Number of PEGs within 90% of the length of their BLASTp best hits (% of row 15)	249 (5%)	648 (6%)	226 (6%)	70 (8%)	4 (2%)
18	Number of f-Tag_0.90_ clusters (% of the grant total, i.e., 9,760)	3,767 (39%)	9,148 (94%)	2,515 (26%)	677 (7%)	148 (2%)
19	Number of consensus protein ID (% of the grant total, i.e., 2,290)	1,227 (54%)	2,209 (96%)	945 (41%)	373 (16%)	88 (4%)

Of all cRNA transcripts in the assemblies, 74% (Trans-ABySS) to 92% (Trinity) contained PEGs (Table 2). As expected, the number of PEGs predicted increased with the size of cRNA assemblies, ranging from 258 (Rockhopper) to 15,705 (Trinity) PEGs (Table 2). Of these, 51% (IDBA-tran) to 90% (Trinity) contained PEGs that ran in the expected direction, indicating that they are likely originated from real transcripts. Similar to the analysis of ncRNA transcripts, PEGs that BLASTp returned with no hits or that showed low-quality alignments with all 10 BLASTp hits were discarded. This quality-filtering step removed from 11% (IDBA-tran) to 23% (Trans-ABySS) of the total number of predicted PEGs from the cRNA assemblies. Even though the assemblies were significantly downsized after filtering away transcripts based on homology search, this has improved the quality of these resultant assemblies and increased the confidence to interpret the community compositions qualitatively and quantitatively.

**Figure 5:**
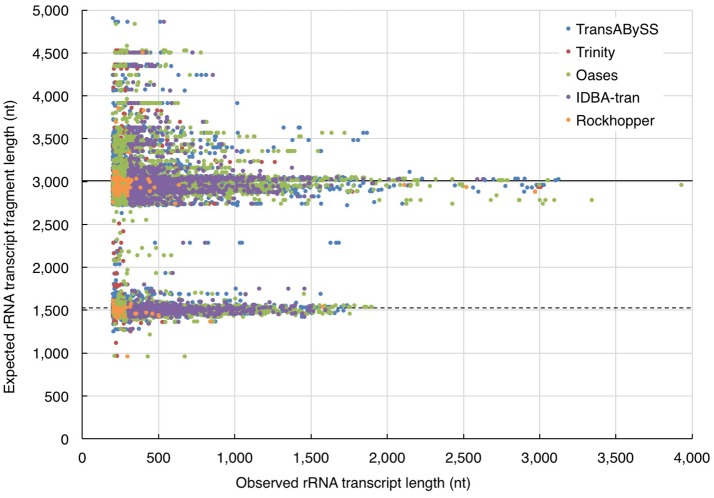
Evaluation of ribosomal RNA (rRNA) transcript length. The length of quality-filtered rRNA transcripts (observed) was compared to the maximum length of qualified BLASTn best hits of the query transcript sequence (expected). The solid and dotted lines denote the average length of large and small subunits (LSU and SSU) of rRNA genes calculated from the all respective qualified BLASTn hits.

**Figure 6:**
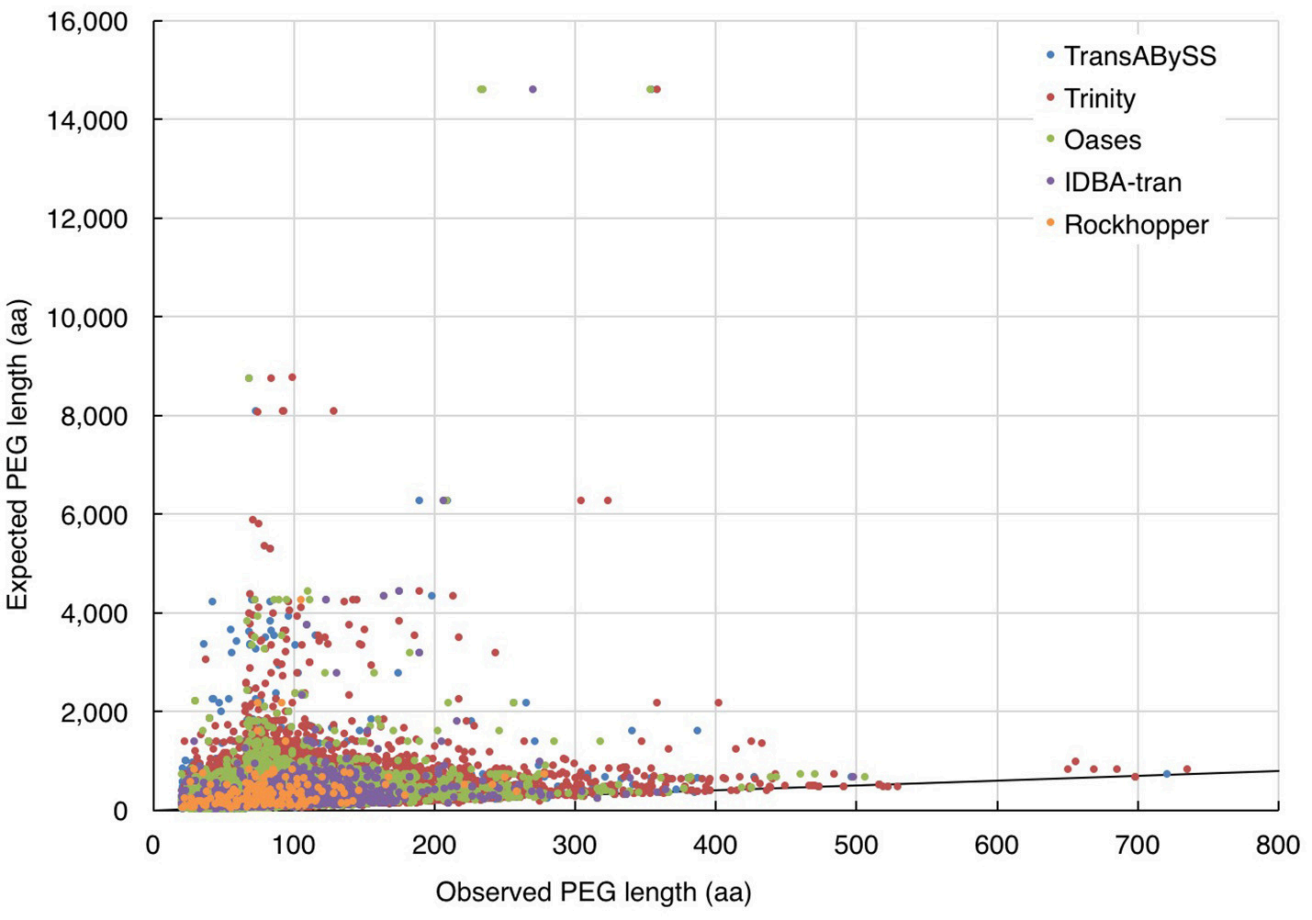
Evaluation of the length of assembled PEGs. The length of quality-filtered PEGs (observed) was compared to the maximum length of qualified BLASTp best hits of the query transcript sequence (expected). The solid line denotes the full-length annotations.

In the absence of ideal reference sets for environmental samples, for evaluating the completeness of transcripts, the maximum length of the qualified BLAST best hits of our transcripts (expected length) was used as a benchmark to further evaluate the transcript length assembled by different assemblers (observed length). The results showed that the majority (≥98%) of the rRNA transcripts in any ncRNA assemblies were shorter than 80% of the expected SSU and LSU lengths, (i.e., ≤ 1,200 nt and ≤ 2,400 nt, respectively; Figure 5, Table 2); whereas, a greater, albeit still low, percentage of PEGs (up to 8%) were assembled to near full length (Figure 6, Table 2).

### Taxonomic composition

The five tested assemblers generated a total of 7,288 t-Tag_0.95_ (Table 2) representing 43 phyla. Only 16 phyla (indeed, all that were detected by Rockhopper) and 35 t-Tag_0.95_ were shared among the five assemblies. It is worth pointing out that these values were limited by the performance of Rockhopper. Otherwise, Trans-ABySS, Trinity, and Oases all detected 38 phyla, while IDBA-tran detected 35 of the 38. However, it is important to note that Trans-ABySS captured 4,506 t-Tag_0.95_ (62% of the grand total), which is ~1,600 t-Tag_0.95_ more than Oases, the second-best-performing assembler (Table 2). Trans-ABySS assembled 191 t-Tag_0.95_ belonging to Candidate divisions vs. 149 by Oases, 78 by IDBA-tran, 72 by Trinity, and 7 by Rockhopper.

**Figure 7:**
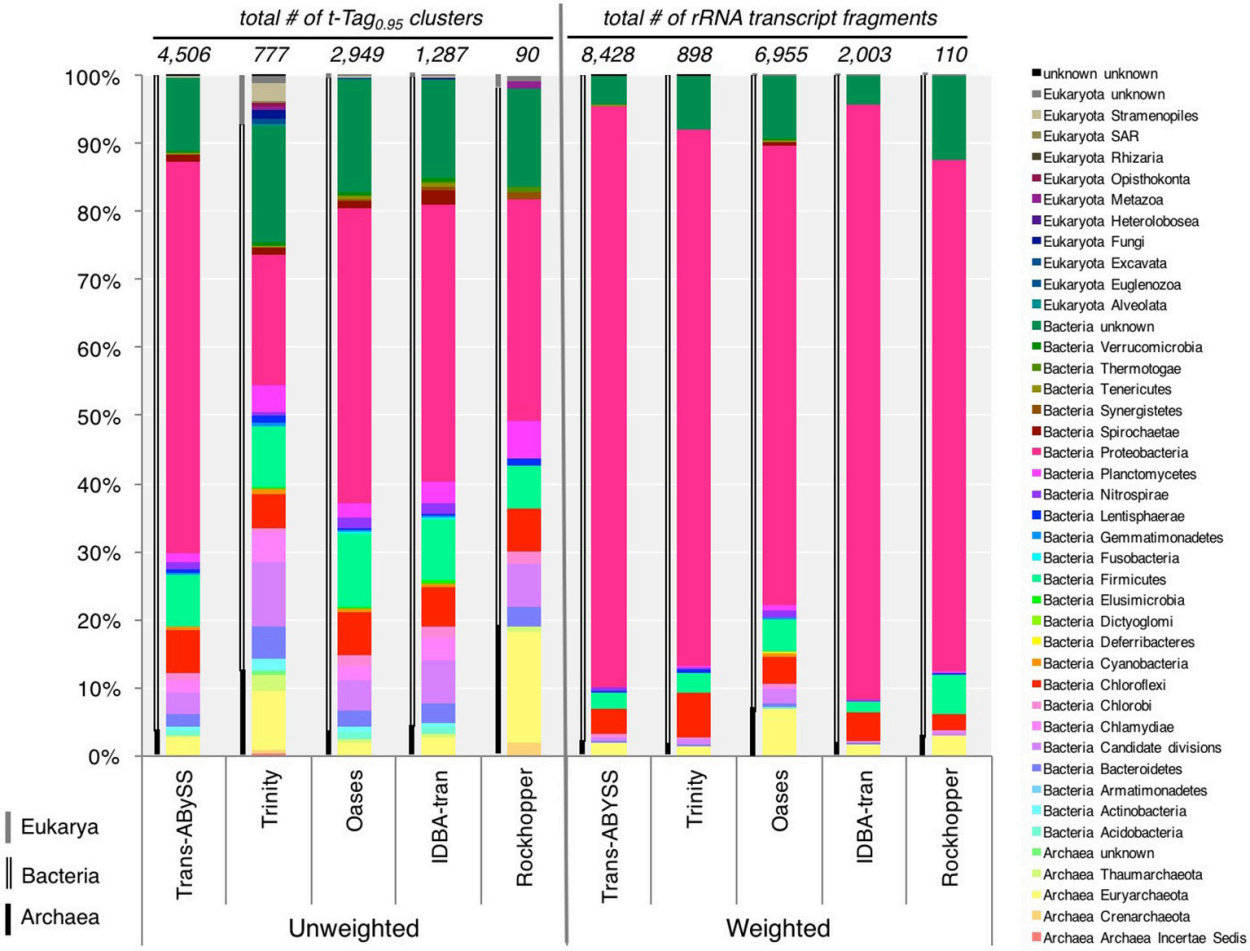
Unweighted and weighted taxonomic compositions revealed by five *de novo* assemblers. For unweighted diversity, distribution of phyla was estimated from the number of the small and large subunits of ribosomal RNA (rRNA) transcripts; whereas coverage, read counts normalized to corresponding transcript length, was used to determine the weighted diversity. The classification of Eukaryota in the database is not as clearly defined as those of Archaea and Bacteria, so taxonomic level immediately below the Domain Eukaryota was taken as “phyla.” Archaeal, bacterial and eukaryal phyla are indicated, respectively, by single black line, double black lines and single gray line to the left of each bar graph. The data is provided in Table S2.

The unweighted (presence or absence of t-Tag_0.95_) and weighted (coverage-based) diversity patterns of all assemblies presented some consistency (Figure 7, Table S2): all three domains of life were present in the studied sample, with Bacteria accounting for at least 96% of t-Tag_0.95_ (unweighted) and the overall coverage (weighted), except in the Rockhopper ncRNA assembly where only 79% of t-Tag_0.95_ (unweighted) was bacteria. The unweighted diversity at the phylum level varied from one assembly to another. Trinity and Rockhopper appear to produce relatively more archaeal rRNA transcripts (more than 10% of t-Tag_0.95_) (Figure 7). A closer examination found that these archaeal t-Tag_0.95_ were represented by less than a hundred transcripts, i.e., fewer than the number of archaeal t-Tag_0.95_ and transcripts detected in the Trans-ABySS and Oases assemblies. Interestingly, the high percentage of eukaryal rRNA transcripts in the Trinity assembly (Figure 7) was indeed supported by the highest number of eukaryal transcripts observed among the assemblies, including five unique eukaryal t-Tag_0.95_ clusters. Though Trinity assembled more transcripts related to eukaryotes, if they were added to Trans-ABySS or Oases assemblies, they would still constitute a minute component (~0.4%) of the overall richness at the phylum level. Considering the highly diverse taxonomic compositions at both the t-Tag_0.95_ and phylum levels, we conclude that Trans-ABySS is the best among the tested algorithms for assembling ncRNA—and more accurately rRNA—reads in metatranscriptomic data.

For each of the assemblies, compared to the unweight diversity pattern, taking into account the abundance record (i.e., weighted diversity) drastically changed the relative distribution of the detected phyla (Figure 7). However, the weighted diversity of the assemblies looked more similar to one another. Proteobacteria largely overtook all of the assemblies, representing 67–87% of the overall community (Figure 7) and 72–89% of the bacterial community (Table 3), whereas the immensely diverse phyla made up a small percentage. The Trans-ABySS rRNA assembly showed that many of the remaining 42 phyla accounted for less than 1% of the total community. These results from ncRNA assemblies confirmed that Proteobacteria, known to dominate the 16S rRNA gene amplicon data (Magnabosco et al., 2014), are indeed the most active microorganisms, and that the rare biosphere detected in the DNA communities (Magnabosco et al., 2014) is not dormant (Table 3).

**Table 3:** Taxonomic composition of bacterial phyla revealed by assembled transcriptomic data (this study) and 16S rDNA V6 amplicon data obtained from the same sample.

**Domain**	**Phylum**	**Trans-ABySS**	**Trinity**	**Oases**	**IDBA-tran**	**Rockhopper**	**16S rDNA V6 amplicons^a^**
		**(weighted)**	**(weighted)**	**(weighted)**	**(weighted)**	**(weighted)**	
Bacteria	Acidobacteria	0.04%	<0.01%	0.16%	0.03%	<0.01%	1.54%
Bacteria	Actinobacteria	0.06%	0.04%	0.11%	0.03%	<0.01%	2.23%
Bacteria	Armatimonadetes	<0.01%	<0.01%	0.01%	<0.01%	<0.01%	ND
Bacteria	Bacteroidetes	0.13%	0.12%	0.39%	0.11%	0.04%	3.34%
Bacteria	Candidate divisions and unknown	4.85%	8.79%	12.23%	4.31%	13.01%	8.42%
Bacteria	Caldiserica	ND	ND	ND	ND	ND	0.04%
Bacteria	Chlamydiae	0.16%	0.10%	0.37%	0.06%	<0.01%	1.57%
Bacteria	Chlorobi	0.37%	0.25%	0.56%	0.27%	0.46%	1.55%
Bacteria	Chloroflexi	3.89%	6.56%	4.28%	4.10%	2.45%	5.32%
Bacteria	Cyanobacteria	0.05%	0.01%	0.71%	0.03%	<0.01%	0.69%
Bacteria	Deferribacteres	0.01%	<0.01%	0.03%	<0.01%	<0.01%	0.86%
Bacteria	Deinococcus	ND	ND	ND	ND	ND	0.49%
Bacteria	Dictyoglomi	<0.01%	<0.01%	<0.01%	<0.01%	<0.01%	ND
Bacteria	Elusimicrobia	0.01%	0.03%	0.09%	0.01%	<0.01%	0.05%
Bacteria	Fibrobacteria	ND	ND	ND	ND	ND	0.08%
Bacteria	Firmicutes	2.38%	2.95%	5.24%	1.57%	5.83%	8.00%
Bacteria	Fusobacteria	0.01%	0.00%	<0.01%	<0.01%	<0.01%	0.02%
Bacteria	Gemmatimonadetes	0.02%	0.00%	0.05%	0.01%	<0.01%	0.40%
Bacteria	Lentisphaerae	0.28%	0.48%	0.25%	0.04%	0.32%	0.53%
Bacteria	Nitrospirae	0.34%	0.22%	1.07%	0.30%	<0.01%	3.17%
Bacteria	Planctomycetes	0.17%	0.27%	0.68%	0.10%	0.15%	0.95%
Bacteria	Proteobacteria	86.96%	80.01%	72.79%	88.92%	77.69%	59.38%
Bacteria	Spirochaetae	0.17%	0.07%	0.50%	0.07%	<0.01%	0.59%
Bacteria	Synergistetes	0.02%	0.02%	0.11%	<0.01%	0.03%	0.04%
Bacteria	Tenericutes	0.03%	0.01%	0.15%	0.01%	<0.01%	0.27%
Bacteria	Thermotogae	0.02%	0.05%	0.17%	0.02%	0.02%	ND
Bacteria	Verrucomicrobia	0.02%	0.01%	0.08%	<0.01%	<0.01%	0.45%

a *Data from Magnabosco et al. (2014). See full citation in the article. Candidate divisions include BRC1, OD1, OP1, OP10, OP11, OP3, OP8, OP9, TA06, TG-1, TM6, TM7, WS3, and WS6*.

*ND, not detected*.

### Functional composition

A total of 9,760 f-Tag_0.90_ clusters, comprised of 2,290 consensus protein IDs, were assembled by the five assemblers (Table 2). Only 68 f-Tag_0.90_ and 65 consensus protein IDs were common to all assemblies. With the largest collection of PEGs successfully assigned with a consensus protein ID, Trinity contributed 990 unique f-Tag_0.90_ (10% of the total) that were not found in other assemblies. The assembly size of cRNA transcripts had a much greater effect on the functional gene profiles than that of ncRNA transcripts on the taxonomic profiles. IDBA-tran and Rockhopper cRNA assemblies would therefore be a poor choice because many PEGs were missing. Trinity is the obvious choice for assembling cRNA reads as it captured the majority of annotations (99% of f-Tag_0.90_ and 96% of consensus protein IDs; Table 2).

**Figure 8:**
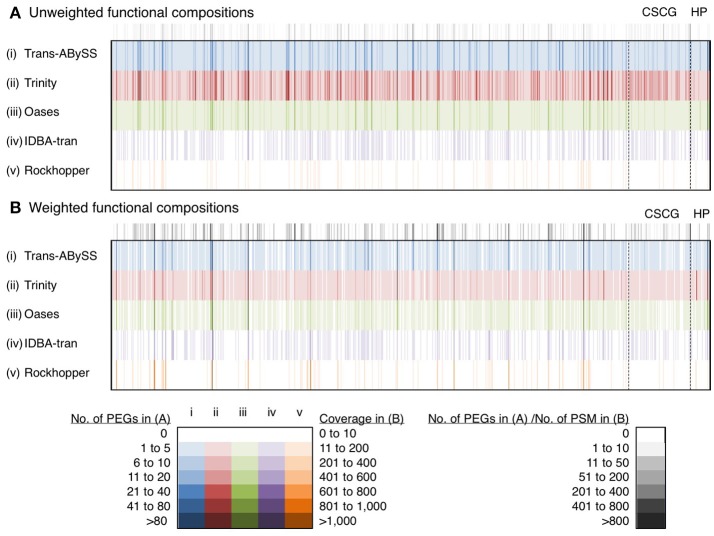
Unweighted and weighted functional diversity profiles of 863 protein IDs. These 863 protein IDs, represented by the color bars, were shared by Trans-ABySS, Trinity and Oases cRNA assemblies. Color intensity indicates the number of protein-encoding genes (PEGs) and coverage (read counts mapped to the parent transcript, then normalized to transcript length) in **(A)** unweighted and **(B)** weighted profiles. Gray scale indicates the number of PEGs and number of peptide-spectral matches (PSM) detected in the metaproteome that were mapped to the **(A)** unweighted and **(B)** weighted profiles, respectively. Abbreviations: CSCG, conserved single-copy gene; HP, hypothetical protein. The data is provided in Table S3.

The performance of these five assemblers to inform functional compositions was compared based on the profiles of 863 consensus protein IDs collectively being detected by Trans-ABySS, Trinity and Oases (Figure 8, Table S3). They were encoded by 3,352 (Trans-ABySS), 6,876 (Trinity), and 2,428 (Oases) f-Tag_0.90_. On average, Trinity assembled two to three times more f-Tag_0.90_ per each of the shared consensus protein IDs. In order to understand whether these f-Tag_0.90_ were genuine variants or they were unlinked fragments potentially originated from the same transcript, we constructed multiple sequence alignments for the f-Tag_0.90_ related to 2-isopropylmalate synthase, and alkyl hydroperoxide reductase subunit C, and their reference sequences in the NCBI nr database. These two enzymes were selected because multiple PEGs related to these two enzymes were assembled by Trans-ABySS, Trinity, and Oases. The alignment of 2-isopropylmalate synthase-related PEGs assembled by Trans-ABySS (10 f-Tag_0.90_), Trinity (21 f-Tag_0.90_), and Oases (7 f-Tag_0.90_) showed that the PEGs overlapped and displayed variations in the amino acid sequence. Examination of alkyl hydroperoxide reductase subunit C-related PEGs of 12 f-Tag_0.90_ from the three assemblers also suggested that many of the f-Tag_0.90_ clusters represent genuinely different variants of the metabolic gene. Of these 863 consensus protein IDs, 89 were related to conserved single-copy genes and 29 were related to hypothetical proteins (see Supplementary Text for discussion).

The presence of transcripts in the cytoplasm is a good indicator of the actual occurrence of represented biochemical reactions; however, their translation into functional proteins or enzymes is governed by post-transcriptional modification and regulation. If the predicted PEGs were translated into proteins, that would further validate the functional inference. The manifestation of PEGs predicted from all transcripts in the metaproteomic data was therefore investigated. In total, 855 cRNA transcripts were detected in the metaproteomic data, which represent 784 f-Tag_0.90_ and 363 consensus protein IDs. For the consensus protein IDs, their expression level, as indicated by the relative abundance of peptide-spectral matches, showed no correlations with the length of PEGs (Figure S1) or the relative abundance of PEGs (Figure S2). Incongruences between transcript and protein expression levels have been documented at the single-cell level and attributed to differences in their decay rates (Moran et al., 2013). Of these expressed proteins, no f-Tag_0.90_ and only hypothetical proteins were detected by all five assemblers. Although Rockhopper and IDBA-tran recovered fewer cRNA transcripts, the cRNA assemblies encompassed a small number of unique f-Tag_0.90_ and consensus protein IDs. Trans-ABySS and Oases recovered similar unique f-Tag_0.90_ (144 and 143, respectively) and consensus protein IDs (103 and 105, respectively). As expected from the large number of cRNA transcripts assembled by Trinity, the Trinity cRNA assembly enabled the detection of the majority of the unique f-Tag_0.90_ (65%) and consensus protein IDs (75%) expressed.

## Discussion

### Reasons to assemble RNA-seq reads

Quality-controlled, unassembled RNA-Seq reads can be interpreted by searching against a large, diverse database (Jiang et al., 2012; Tveit et al., 2013) such as RDP and SILVA for ribosomal RNA, and Pfam and Swiss-Prot for PEGs, but the short read-length (~100 bp) limits the confidence of the gene annotation. It is less a problem if more stringent search criteria are used to assign short reads with a taxonomic or functional identity and, if the search is against a more specific reference set, e.g., genomes (Stewart et al., 2011), metagenomes from the same sample (Hultman et al., 2015) and, single-cell genomes (Embree et al., 2014). Pipelines exist to facilitate such (meta)genomic and (meta)transcriptomic data integration by aligning RNA-Seq reads to genomic templates, for example, IMP (Narayanasamy et al., 2016) and ATLAS (White et al., 2017). Despite the growing number of published microbial genomes that are generated from isolates (Dam et al., 2012), metagenomes (Castelle et al., 2013) and single-cell genomes (Rinke et al., 2013), only 1–2% of these genome-defined species have been documented by environmental 16S rRNA gene sequences. This number of genomes is then inadequate for annotating unassembled RNA-Seq reads. If one uses contiguous sequences, or contigs, constructed from (meta)genomic assembly for reference-based annotation of RNA-Seq reads, there are two main caveats: a metagenomic assembly may be prone to annotation errors, and rarely is an assembly large enough to capture all organisms in the studied community. As a result, a yet-to-be-quantified percentage of unassembled, short RNA-Seq reads may either fail to be annotated due to poor matching with the reference sequences, or may be assigned with a wrong identity.

One of the ways to enhance our RNA-Seq data annotation is to increase query sequence length. Nowadays, longer read lengths can be achieved by using recently launched preparation kits (e.g., Illumina MiSeq Reagent Kit v3, Illumina TruSeq Synthetic Long-Read Library Prep Kit) or other sequencing technologies [e.g., 454, Pacific-Biosciences SMRT and Oxford Nanopore (Conesa et al., 2016)]. Alternatively, post-quality-controlled reads can be assembled into contigs or even near-full length transcripts, which can then be used for gene prediction and annotation. For data from environmental samples, guided-assembly by first aligning reads to a reference is not always preferred due to the lack of high-quality reference partial or complete genomes for uncultivated organisms. Unlike guided-assembly, *de novo* assembly reconstructs transcripts directly from RNA-Seq data, and therefore does not suffer from errors inherited from poor reference genomes derived from (meta)genome assembly that are poorly annotated. In the absence of *a priori* templates as used in guided-assembly, *de novo* assembly also allows the detection of novel isoforms (Garber et al., 2011).

### k-mer size and transcript expression level

As transcript expression levels within one single cell spans few orders of magnitude (Bernstein et al., 2002), the range for a community is likely to be as wide, if not wider, because members in a diverse microbial community may occur at different abundances, and each population may display different degrees of cellular activity. It has been suggested that transcripts of higher expression levels are better represented when longer k-mer sizes are used, whereas lowly expressed transcripts are better represented when shorter k-mer sizes are used (Robertson et al., 2010; Schulz et al., 2012). Assemblers that create multiple, independent assemblies from different k-mer sizes, and merge them afterwards are therefore expected to better capture the few-orders-of-magnitude wide dynamic range of transcript abundance. In contrast to this notion, the expression range of transcripts in Trans-ABySS and Oases assemblies, created by multiple k-mers, were relatively low when compared to the assemblies generated by the other three assemblers (Figure 2). The expression range of cRNA transcripts assembled by Oases (0–1,692) was in agreement to the range (0–1,600) reported in a study that applied a similar approach on a dataset at least 10 times larger (Baker et al., 2013). Therefore, the relatively lower expression range detected by Oases assemblies in this study was not likely due to insufficient RNA-Seq reads. Although IDBA-tran assembled fewer transcripts (Table S1), it detected a considerable range of transcript expression levels (0–243,188) (Figure 2). Like Trans-ABySS and Oases, IDBA-tran also uses multiple k-mer sizes for assembly. However, unlike Trans-ABySS and Oases, the final assembly is dependent on the previous assemblies that were constructed progressively with increasing k-mer sizes. Moreover, the assemblies generated from single k-mers displayed a wide transcript expression range, with 0–218,655 for Trinity assemblies and 28–341,743 for Rockhopper assemblies (Figure 2). Hence, the coverage distribution plots showed that a larger-size assembly (e.g., Trans-ABySS, Trinity and Oases) is more likely to capture more of the transcripts that occurred at lower expression levels (<100 coverage), and the use of multiple k-mers or single k-mers has no clear influence on the expression range of transcripts detected in assembled metatranscriptomes.

### Completeness and continuity of metatranscriptomic assemblies

N50 and N90 are defined as the contig lengths that mark 50 and 90%, respectively, of the total assembled bases in contigs longer than such length. These indices have commonly been used to assess the quality of metagenomic assemblies based on the assumption that better assemblies produce longer contiguous sequences. Generally speaking, the N50 values suggested that assemblies from multiple k-mers (Trans-ABySS, Oases and IDBA-tran) tend to generate more and longer transcripts than those from single k-mers (except the Trinity cRNA assembly) (Table S1), whereas the N90 values are not informative at all because the majority of all assemblies consisted of short transcripts. Our results supported the argument that using N50 and N90 to evaluate RNA-Seq assemblies is not as meaningful due to significantly narrower transcript length distribution of transcripts (Li et al., 2014). Transcript continuity is evaluated based on the concept that, with the same set of RNA-Seq reads, the assembly with the greatest number of reads represented in the least number of contigs will likely be the best, if not the true, assembly (Li et al., 2014). Transcript continuity is therefore a more useful measure than the traditional indices when assessing whether a metatransciptomic assembly is composed of many fragmented contigs.

The higher RSEM-EVAL scores of cRNA assemblies than that of ncRNA assemblies (Figure 4) indicated that constructing continuous transcripts from cRNA reads is more achievable by various *de novo* transcriptomic assemblers than from ncRNA reads or all reads (i.e., ncRNA-dominated data). In other words, assembling ncRNA reads that are primarily rRNA genes is more challenging. One possible explanation is the presence of micro-heterogeneities. rRNA genes are conserved across all three domains of life. Variations in the rRNA genes of diverse microorganisms, while useful for differentiating lineages, however present a computational problem because taxonomic diversity and complexity of a sample introduce micro-heterogeneities. Hypervariable regions in the 16S rRNA gene increases the number of possible paths by increasing bubbles and frayed ropes, which can “trick” the assemblers. Micro-heterogeneities greatly increase the complexity of the *de Bruijn* graphs and/or halt the extension of a growing contig. Therefore, the nature of input sequences is the *a priori* factor to consider when choosing an assembler. Some assembly algorithms such as EMIRGE (Miller et al., 2011) and, more recently, REAGO (Yuan et al., 2015) are designed for assembling SSU genes and quantifying their relative abundances. EMIRGE is popular and produces reproducible results (Miller et al., 2013). However, these algorithms were tested in this study because their performance in assembling LSU, which is more abundant than SSU in total RNA, has not been evaluated. Among the five assemblers tested in this study, Trans-ABySS and Trinity generated cRNA assemblies with similarly high continuity. The ncRNA assembly of the highest continuity was generated by Trans-ABySS.

All five transcriptomic assemblers used in this studied were designed and tuned to analyze data from a single species or mammalian cell line. Their ability to assemble transcripts of near-complete length has been reported. However, a large proportion of incomplete transcripts were observed in our ncRNA and cRNA assemblies (Figures 5, 6). With a mixture of rRNA gene sequences from closely related species and functional gene sequences from distantly related species, i.e., a higher data complexity, the performance of all assembler on metatranscriptomic data was less optimal than their performance on transcriptomic data from pure cultures (Celaj et al., 2014). Our single-end reads are unsuitable for testing the recent algorithms developed for assembling metatranscriptome data, which require paired-end reads (Leung et al., 2014; Ye and Tang, 2016). Nonetheless, the assembly of mouse gut RNA-Seq data by transcriptomic assemblers Trinity and Oases was shown to have better functional annotation rates than that of metatranscriptomic assembler IDBA-MT (Celaj et al., 2014). If transcriptomic assemblers are as good as metatranscriptomic assemblers for assessing a highly diverse and complex community, increasing the sequencing depth would likely increase the completeness of assembled transcripts. However, increased RNA sequencing depth requires greater computational power.

### Impact on taxonomic and functional compositions

Assemblies are commonly evaluated primarily based on the physical properties of the assembled transcripts. However, taxonomic and functional diversity patterns are the strong foundations in environmental microbiology, from which further investigations (e.g., microbial biogeography, biogeochemical networks, microbial food-webs) can be pursued. Few studies have compared the performance of different transcriptomic assemblers on RNA-Seq data. Such a comparison performed on a mixed-species microbial community is even scarcer.

Analyzing t-Tag_0.95_ in metatranscriptomes is useful for elucidating the relative distribution of active archaeal, bacterial and eukaryal members within a sample because primer bias is less significant compared to hybridization-based detection methods (e.g., reverse-transcription quantitative PCR and microarray). Unlike sequences recovered from PCR-based methods, these t-Tag_0.95_ do not cover the same region but encompass the entire SSU or LSU genes instead. As these t-Tag_0.95_ were not assembled to the (ideal) full length, the more identified t-Tag_0.95_ clusters indicate a better coverage of the overall rRNA gene diversity. And at least in the case of the SSU t-Tag_0.95_, the 200-nt minimum length implies that the clusters contain both hypervariable and conserved regions, which allow more reliable taxonomic assignment than unassembled short reads do. Therefore, an assembler is preferred if it captures the most phylum-level diversity and the greatest number of t-Tag_0.95_ possible. Trans-ABySS is superior because it detected 39 phyla, capturing 4,506 t-Tag_0.95_ (62% of the grand total) in the ncRNA assembly (Table 2, Figure 7). These ncRNA transcripts cover a good range of size (Figure 3, Figure 5) and expression (Figure 2). Trans-ABySS also assembled 191 t-Tag_0.95_ belonging to Candidate divisions. Among the Candidate divisions, “*Candidatus* Desulforudis” was represented by six t-Tag_0.95_ (20 transcripts) in the Trans-ABySS assembly vs. four t-Tag_0.95_ (8 transcripts) in the Oases assembly, one in the Trinity assembly and none in the remaining two. “*Candidatus* Desulforudis” and its close relatives have been detected exclusively in many subsurface environments (Gihring et al., 2006; Chivian et al., 2008; Alawi et al., 2011; Itävaara et al., 2011; Brazelton et al., 2012; Jungbluth et al., 2013; Tiago and Veríssimo, 2013; Osburn et al., 2014; Robador et al., 2015; Purkamo et al., 2016). The choice of assemblers is crucial for providing the first empirical evidence that this spore-forming sulfate-reducing bacterium, putatively endemic in the subsurface, is transcriptionally active in the subsurface.

**Figure 9:**
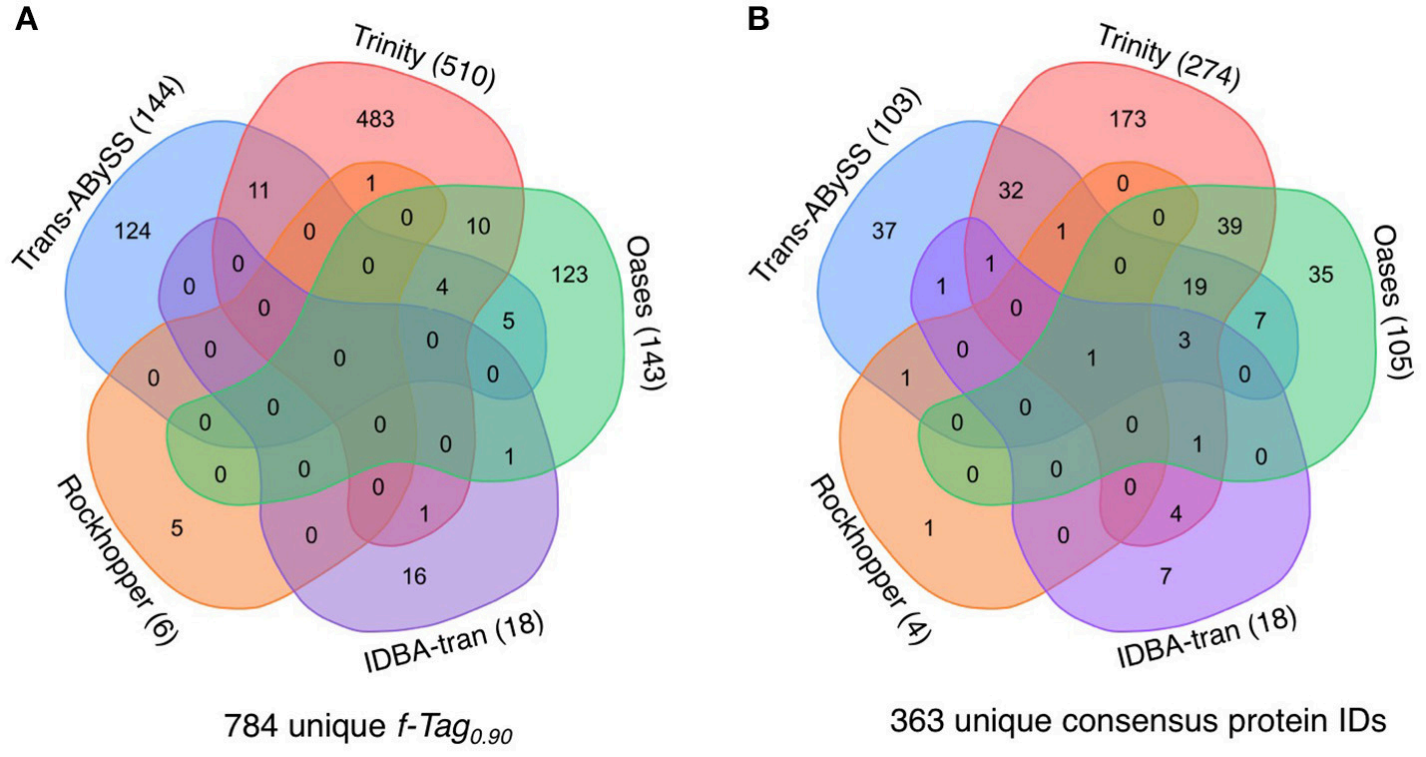
Expression of proteins predicted from cRNA transcripts. The Venn diagram illustrates the shared and distinct **(A)** f-Tag_0.90_ of cRNA transcripts and **(B)** consensus protein IDs from the analysis of the metaprotoemic data. All cRNA transcripts generated from the five cRNA assemblies were compiled into one single peptide datase for protein identification. The Venn diagram was generated using the tool at http://bioinformatics.psb.ugent.be/webtools/Venn/.

For assembling cRNA reads, Trinity outperformed other assemblers by reconstructing 9,148 f-Tag_0.90_, including 990 unique f-Tag_0.90_. These cRNA transcripts showed reasonable range of size (Figures 3, 6) and expression (Figure 2). They coded for 2,209 consensus protein ID (96% of all predicted consensus protein IDs) (Table 2). Analysis of metaproteomic data validated the functional inference of approximately 12% of the predicted proteins (Figure 9, Table S4). To further illustrate the importance of obtaining good cRNA assemblies, we compared the nitrogen, sulfur, and carbon metabolisms revealed by the best two cRNA assemblies generated by Trinity and Trans-ABySS. Biogeochemical cycling of nitrogen, sulfur, and carbon in the deep subsurface has strong implications on the questions of how the subsurface environment shapes the light-independent microbial community and vice versa. As reported in by Lau et al. (2016), the Trinity cRNA assembly showed the dominant pathway of N, S, and C metabolisms in the studied borehole was denitrification, sulfur oxidation, and Calvin-Benson-Bassham cycle for CO_2_ fixation, respectively. Although the Trans-ABySS cRNA assembly revealed similar results, this assembly missed many lowly expressed transcripts detected in Trinity cRNA assembly (black ellipses in Figure S3). Hence, the metabolic network shown by Trans-ABySS cRNA assembly lacked details when compared to the one shown by Trinity cRNA assembly. For example, the PEGs coding for the key enzymes hydroxylamine oxidase (HAO) in anaerobic ammonia oxidation, acetate kinase (ACK) and phosphate acetyltransferase (PTA) for the conversion between acetyl-CoA and acetate were absent. The coverage ratio of PEGs coding for reverse-type dissimilarly sulfite reductase to that for dissimilarly sulfite reductase was remarkably different between the two cRNA assemblies. These two enzymes are indicators for sulfur oxidation and sulfate reduction, respectively. A ratio of 1:1 in Trinity cRNA assembly vs. 1:5 in Trans-ABySS cRNA assembly would suggest a different story about the subsurface sulfur cycle. Only by the analyses of PEGs coding for adenylylsulfate reductase (APR) and sulfate adenylyltransferase (SAT) could the dominance of sulfur oxidation activity in both cRNA assemblies be confirmed.

## Conclusion

Metatranscriptomics of total RNA (i.e., non-rRNA-depleted RNA) informs both taxonomic and functional compositions of the active microbial community in environmental samples. This information can be extracted by *de novo* assembly and does not require (meta)genomic references. This study showed that assemblies of RNA-Seq data generated by different *de novo* transcriptomic assemblers vary in assembly size, length, and coverage distributions of the assembled transcripts, taxonomic and functional diversity patterns, and ultimately, the metabolic network. Of the five tested assemblers, Trans-ABySS, and Trinity are the recommended assemblers for ncRNA and cRNA reads, respectively. Choosing a suitable assembler and careful evaluation of the assemblies will enable a comprehensive view of the *in situ* metabolic landscape, and will generate quality expression profiles for differential expression analyses. Our study provides useful aspects for designing metatranscriptomic experiments.

Through *de novo* assembly of metatranscriptomic data, active microorganisms from 43 phyla across all three domains of life were detected in the studied sample, which makes this sample one of the highest diversity samples reported for deep subsurface environments and ecosystems with extreme conditions. With modifications, the bioinformatics and evaluation approach presented here should be applicable to analyzing other terrestrial and oceanic samples that may show a more complex transcriptional network (Aylward et al., 2015; Yergeau et al., 2018).

## Author contributions

ML: conceived the study. ML, RH, YO, MY, and TO: assembled metatranscriptomic data using different algorithms and performed post-assembly statistics analysis. ML: compared the assemblies. AB and ML: compiled the source codes for public release. All authors discussed the results and commented on the manuscripts drafted by ML.

### Conflict of interest statement

The authors declare that the research was conducted in the absence of any commercial or financial relationships that could be construed as a potential conflict of interest.
