# Supplementary material for: Taxonomic and Functional Compositions Impacted by the Quality of Metatranscriptomic Assemblies
Source: Front Microbiol. 2018 Jun 20;9:1235. doi: 10.3389/fmicb.2018.01235 (PMC6019464; doi:10.3389/fmicb.2018.01235)
Supplement: Supplementary file 9 [file Data_Sheet_4.docx]

Supplementary Material

Taxonomic and functional compositions impacted by the quality of metatranscriptomic assemblies

Maggie C.Y. Lau^1^*, Rachel L. Harris^1^, Youmi Oh^2,a^, Min Joo Yi^3^, Aida Behmard^4,b^ and Tullis C. Onstott^1^

*** Correspondence:** Maggie C.Y. Lau: [maglau@princeton.edu](mailto:maglau@princeton.edu)

# Supplementary Tables

Table S1. Summary of de novo metatranscriptomic assemblies of non-coding (nc), coding (c) and all high-quality RNA reads generated by assemblers (i) Trans-ABySS, (ii) Trinity, (iii) Oases, (iv) IDBA-tran, and (iv) Rockhopper.

Table S2. Unweighted and weighted taxonomic profiles generated by five de novo transcriptomic assemblers. The data was used to create Figure 8.

Table S3. Unweighted and weighted functional diversity profiles of 863 protein IDs. The data was used to create Figure 9.

Table S4. Expression of f-Tag_0.90_ (left) and consensus protein IDs (right) predicted from cRNA transcripts. The data was used to create Figure 9.

Table S5. Glossary of enzyme codes in Figure S3.

**Supplementary Figures**

**Figure S1. Relationship between the length of protein-encoding genes (PEG) and the relative protein expression level data.** The expression level of the encoded protein was the percentage of peptide-spectral matches (PSM) relative to the total PSM detected in the run. Filtered data from technical replicate runs were plotted separately.

**Figure S2. Relationship between the transcript and protein expression level of protein-encoding genes (PEG).** The expression level of a PEG was expressed as its mean base coverage relative to the sum of mean base coverage of all detected cRNA transcripts. The expression level of the encoded protein was the percentage of peptide-spectral matches (PSM) relative to the total PSM detected in the run. Filtered data from technical replicate runs were plotted separately.

**Figure S3. Comparison of metabolic pathways detected in Trinity versus Trans-ABySS cRNA assembly.** Pathways of nitrogen, sulfur and carbon metabolisms are enclosed in blue, yellow and brown envelopes, respectively. Black and grey solid lines indicate reactions mediated by enzymes, whereas black dotted lines in the N cycle indicate abiotic reactions. Red eclipses represent enzymes encoded by PEG transcripts detected in both Trinity and Trans-ABySS cRNA assemblies, whereas black eclipses represent enzymes encoded by PEG transcripts detected in Trinity cRNA assembly alone. Abbreviations: CBB, Calvin-Benson-Bassham cycle or reductive pentose phosphate cycle; rTCA reverse tricarboxylic acid cycle; 3-HP/4-HB, 3-hydroxypropionate/4-hydroxybutyrate (3-HP/4-HB) cycle; WL, Wood-Ljundahl or reductive acetyl-CoA pathway; MEGEN, methanogenesis (downward direction); ANME, anaerobic methane oxidation (upward direction); EMP, Embden-Meyerhof-Parnas glycolysis (downward direction); GLUCO, Gluconeogenesis (upward direction); SERINE, formaldehyde assimilation via serine pathway; 3PG, 3-phosphoglycerate; DNRA, dissimilatory nitrate reduction to ammonia; ANAMMOX, anaerobic ammonium oxidation. This figure shares the same template as Figure 1 in Lau et al. 2016. The list of enzymes is available in Table S5.

**Supplementary Text**

*Potential RNA contamination*

It would have been ideal to set up a control filter alongside with the sample at the site, but such control was not available. Typical deep fracture fluid is anoxic and has a reducing potential. In order to diagnose air contamination during sampling, on the day of filter collection, water flowing out of the effluent channel of the filter housing was tested to confirm that its temperature and Eh were similar to that of the water going into the filter. Δ^13^C and δ^13^C analyses of CH_4_, DIC, bacterial PLFA samples collected during the same visit showed that these carbon species had “old” carbon (Simkus et al., 2016). These results indicated that there were very little or negligible inputs of modern carbon, strongly suggesting that there was little or no contamination from drilling or service water.

DNA contamination in extraction and sequencing reagents has been shown to impact significantly the interpretation of microbial composition in low-biomass samples (Salter et al., 2014; Glassing et al., 2016). In contrast, the impact of RNA contamination in extraction and sequencing on RNA-Seq data has not been studied. Identify and quantity of RNA contamination was not performed for the studied samples. Assuming RNA contaminants were originated from the genera detected by DNA-based analyses (Salter et al., 2014; Glassing et al., 2016), we detected ncRNA transcripts belonging to 35 genera of potential contaminants. They altogether were present at 1.1% (29 genera) in the Trans-ABySS ncRNA assembly, 0.4% (11 genera) in the Trinity ncRNA assembly, 2.3% (27 genera) in the Oases ncRNA assembly, 13.0% (15 genera) in the IDBA-tran ncRNA assembly, and 0.4% (2 genera) in the Rockhopper ncRNA assembly. If our assumption that RNA contaminants came from the same genera as those detected by DNA-based analyses was correct, the contamination level was relatively low in all ncRNA assemblies except the Rockhopper’s. These results indicated that the choice of assembly affects the interpretation of these potential contaminants. The lack of procedural blank for examining RNA contaminants may explain the low occurrence of contaminant genera that were reported in DNA, and in the extraction and sequencing reagents that were not used in our experiment. More likely explanations are the large number of microbial cells collected on the filter, and the collected community were active instead of dormant.

One may concern also about potential microbial contamination from service water and drilling fluid. We therefore also estimated the relative abundance of 79 genera that have been detected in service water and drilling fluid in mines (Magnabosco et al., 2014b). They were present at 11.4% (24 genera) in the Trans-ABySS ncRNA assembly, 5.8% (6 genera) in the Trinity ncRNA assembly, 1.9% (17 genera) in the Oases ncRNA assembly, 16.5% (11 genera) in the IDBA-tran ncRNA assembly, and 2.1% (1 genus) in the Rockhopper ncRNA assembly. However, it was not certain whether these genera were true contaminants (Magnabosco et al., 2014b) because service water and drilling fluid were often sourced from the groundwater in the mining area. Since the metabolic processes reconstructed from this RNA-Seq data and the isotopic geochemistry data have presented a coherent story (Lau et al., 2016), in this case it is very likely that the genera similar to those reported in service water and drilling fluid were indigenous deep surface microorganisms.

Unlike very-low-biomass samples, the dominance of active cells in the studied sample over potential RNA contaminants in reagents render the impact of RNA contamination in our RNA-Seq data. It is anticipated that RNA contamination in extraction and sequencing reagents would affect significantly the quality and quality of RNA-Seq data. Further investigations are required to compare systematically methods to minimize, quantify, and filtering away RNA contaminants in RNA-Seq data. For removing RNA contaminant sequences from 16S rRNA amplicon and shotgun sequencing data, we may need different practices for these two different types of data.

*Protein expression of predicted PEGs with unknown function*

A considerable portion (24-26%) of the PEGs detected in cRNA assemblies, supported by 21-35% of coverage, was closely related to hypothetical proteins (denoted by HP in Figure 8). Although the functions of these proteins have not yet been determined, peptides of PEGs were identified in metaproteome data, suggesting that some of the hypothetical proteins could be enzymes or co-factors essential for living in the subsurface. Notably, about 70 low-quality PEGs discarded due to their low BLASTp scores in the homology search matched to the MS/MS spectral data. The subterranean aquatic environment is one of the habitats commonly searched for uncultivated microorganisms or often referred as “microbial dark matter” (Rinke et al., 2013). These low-quality PEGs might be real proteins of novelty and further investigation is required to test this hypothesis.
