# Supplementary figures and images for: Taxonomic and Functional Compositions Impacted by the Quality of Metatranscriptomic Assemblies
Source: Front Microbiol. 2018 Jun 20;9:1235. doi: 10.3389/fmicb.2018.01235 (PMC6019464; doi:10.3389/fmicb.2018.01235)

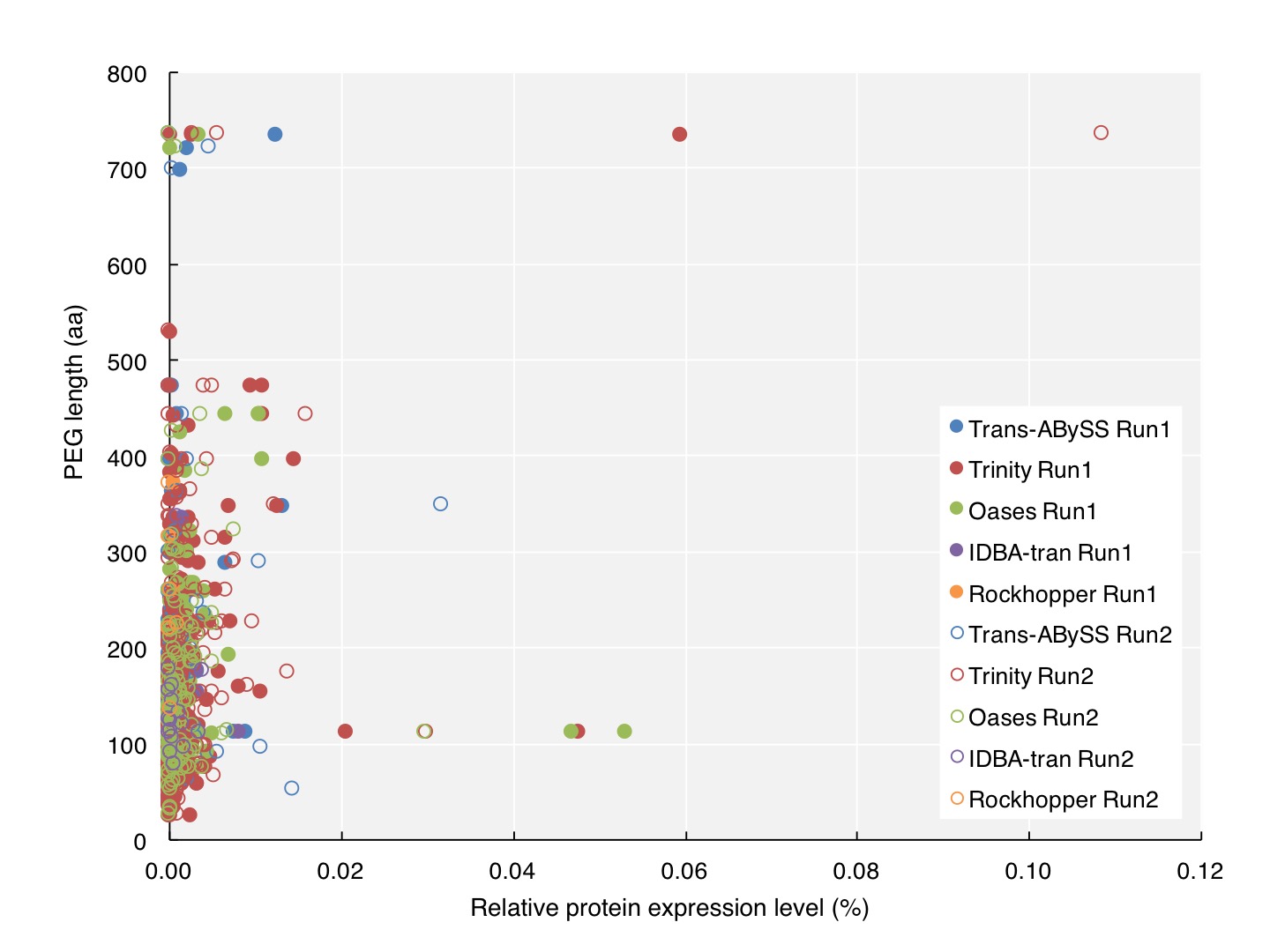

Supplement: Supplementary file 10 [file Image_1.JPEG]

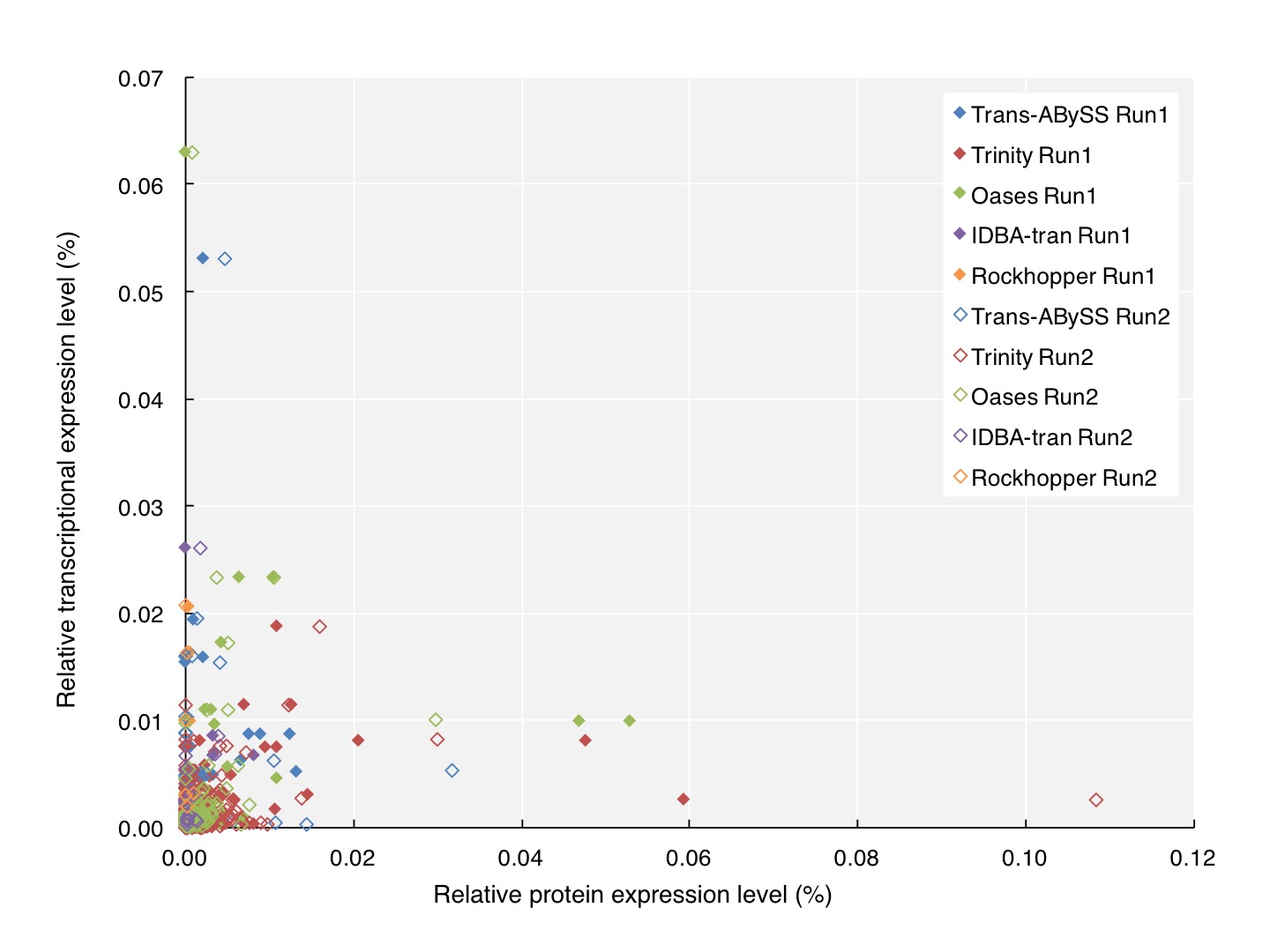

Supplement: Supplementary file 11 [file Image_2.JPEG]

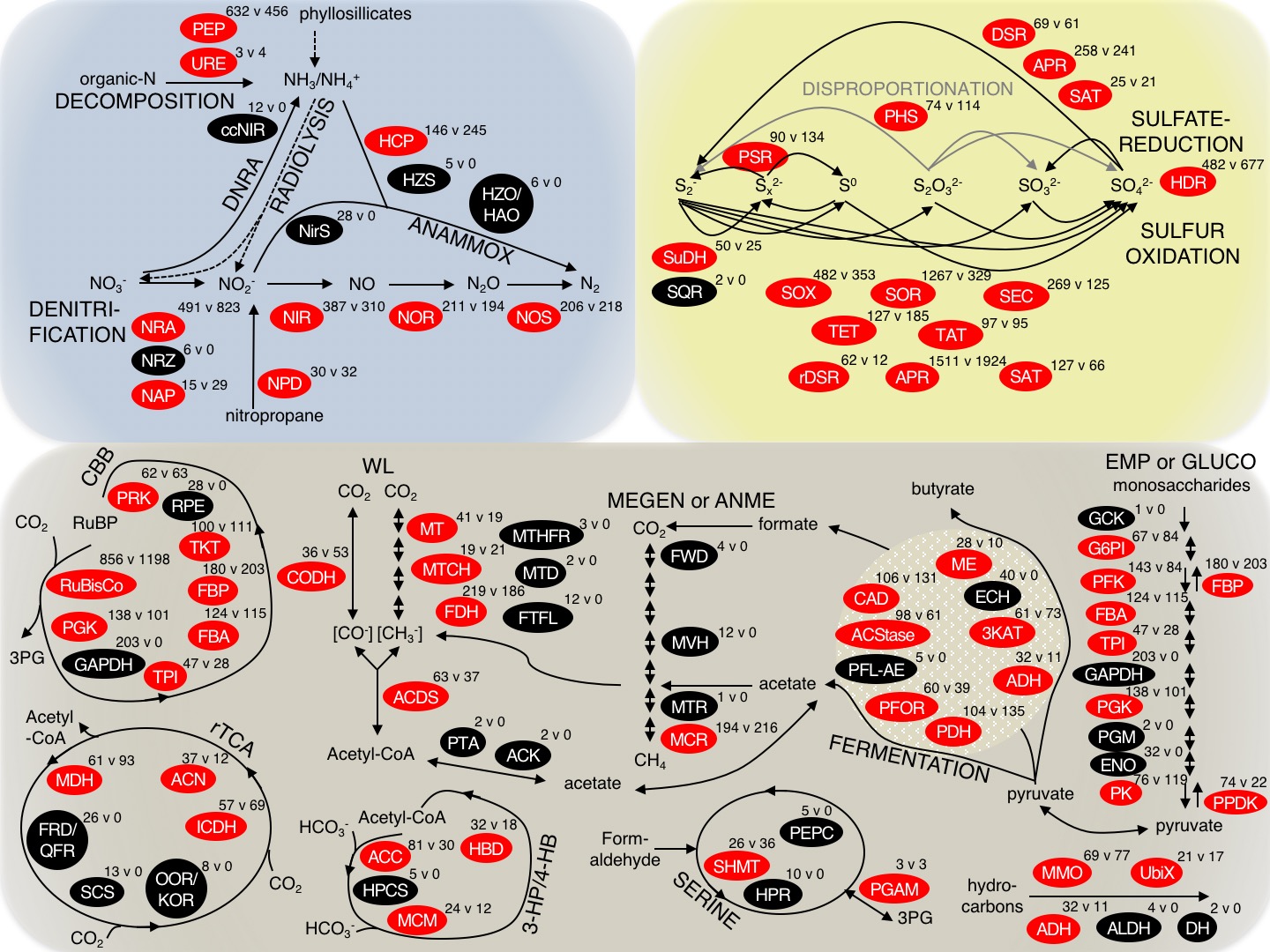

Supplement: Supplementary file 12 [file Image_3.JPEG]
